# Supplementary material for: Exogenous CFH Modulates Levels of Pro-Inflammatory Mediators to Prevent Oxidative Damage of Retinal Pigment Epithelial Cells with the At-Risk CFH Y402H Variant
Source: Antioxidants (Basel). 2023 Jul 31;12(8):1540. doi: 10.3390/antiox12081540 (PMC10451625; doi:10.3390/antiox12081540)
Supplement: Supplementary file 1 [file antioxidants-12-01540-s001.zip › antioxidants-2476960-supplementary.pdf]

Exogenous CFH Modulates Levels of Pro-Inflammatory Mediators to Prevent Oxidative Damage of Retinal Pigment Epithelial Cells with the At-Risk CFH Y402H Variant

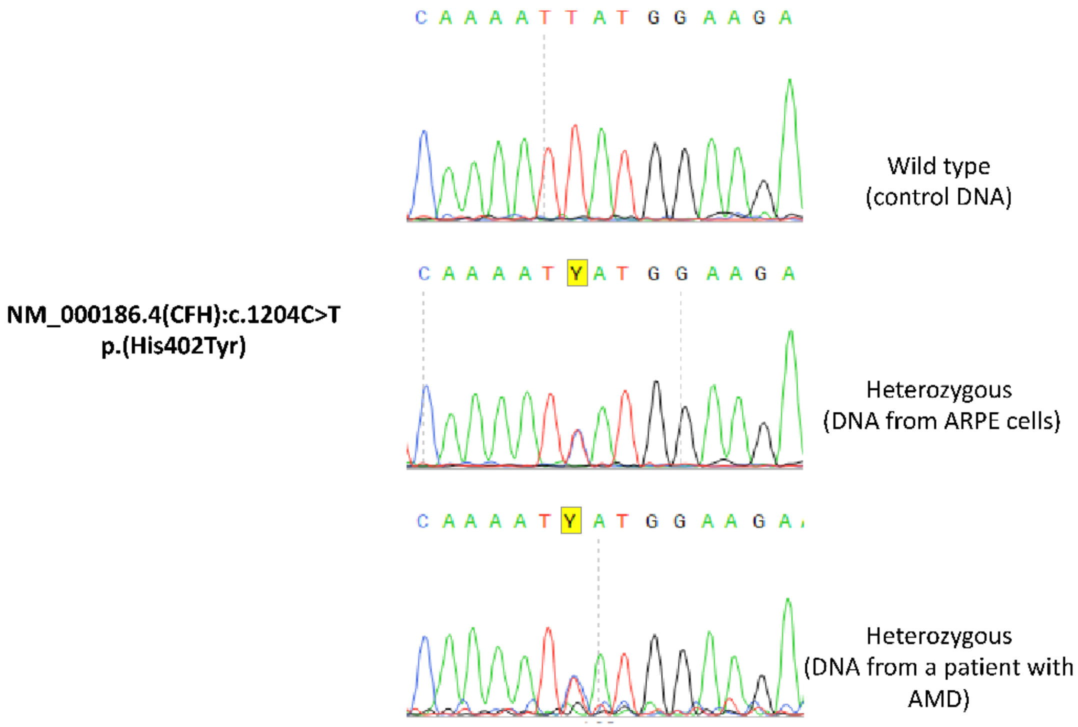

Figure S1. Partial Sanger sequencing of the CFH gen. Sanger sequencing from a wild-type individual, heterozygous c.1204T>C variant in ARPE-19 cells, and heterozygous c.1204T>C from a patient with AMD.
